# The lateral parabrachial nucleus is actively involved in the acquisition of fear memory in mice

**DOI:** 10.1186/s13041-015-0108-z

**Published:** 2015-03-27

**Authors:** Masaru Sato, Mariko Ito, Masashi Nagase, Yae K Sugimura, Yukari Takahashi, Ayako M Watabe, Fusao Kato

**Affiliations:** Department of Neuroscience, Jikei University School of Medicine, Tokyo, 105-8461 Japan; Department of Anesthesiology, Jikei University School of Medicine, Tokyo, 105-8461 Japan; Precursory Research for Embryonic Science and Technology (PRESTO), Japan Science and Technology Agency, Kawaguchi, Saitama 332-0012 Japan; Nagoya University Graduate School of Medicine, Nagoya, 466-8550 Japan

**Keywords:** Fear conditioning, Associative learning, Nociception, Unconditioned stimulus, Amygdala, Channelrhodopsin, Virus

## Abstract

**Background:**

Pavlovian fear conditioning is a form of learning accomplished by associating a conditioned stimulus (CS) and an unconditioned stimulus (US). While CS–US associations are generally thought to occur in the amygdala, the pathway mediating US signal processing has only been partially identified. The external part of the pontine lateral parabrachial nucleus (elPB) is well situated for providing US nociceptive information to the central amygdala (CeA), which was recently revealed to play a primary role in fear acquisition. Therefore, we manipulated the elPB activity to examine its role in the regulation of fear learning.

**Results:**

First, we transiently inactivate the elPB during the acquisition of fear memory. Mice received bilateral elPB injections of the GABA_A_ agonist muscimol (MUS) or phosphate-buffered saline (drug control), with bilateral misplacement of MUS defined as a placement control group. After the injection, mice were conditioned with a pure tone and foot-shock. On a memory retrieval test on day 2, the freezing ratio was significantly lower in the MUS group compared with that in the drug control or placement control groups. A second retrieval test using a pip tone on day 4 following *de novo* training on day 3, resulted in significant freezing with no group differences, indicating integrity of fear learning and a temporary limited effect of MUS. Next, we examined whether selectively activating the elPB-CeC pathway is sufficient to induce fear learning when paired with CS. Mice with channelrhodopsin2 (ChR2) expressed in the elPB received a pure tone (CS) in association with optical stimulation in the CeA (CS-LED paired group). On the retrieval test, CS-LED paired mice exhibited significantly higher freezing ratios evoked by CS presentation compared with both control mice receiving optical stimulation immediately after being placed in the shock chamber and exposed to the CS much later (immediate shock group) and those expressing only GFP (GFP control group). These results suggest that selective stimulation of the elPB-CeC pathway substitutes for the US to induce fear learning.

**Conclusions:**

The elPB activity is necessary and sufficient to trigger fear learning, likely as a part of the pathway transmitting aversive signals to the CeA.

## Background

The amygdala plays a key role in fear learning by attaching emotional value to various sensory inputs. The neural mechanism underlying this associative learning of conditioned stimulus (CS) and an unconditioned stimulus (US) has been intensively studied using aversive signals, such as electrical foot-shock, as the US [1-3]. Nociceptive peripheral nerve activation induced by an electrical shock is speculated to serve as a US signal that enables an emotionally neutral CS to elicit defensive responses after associative plasticity occurs in amygdala networks. This view is supported by recent studies indicating that a selective optogenetic activation of subcutaneous C-fibers induces place aversion [4,5].

Thus, an unanswered question is through which pathways does the amygdala receives such nociception-related US information in fear learning. Of the brain areas involved in pain signaling, the periaqueductal grey (PAG) and the anterior cingulate cortex (ACC) have been shown to regulate fear learning [6,7]. However, because of the indirect nature of the connections from the spinal cord to the ACC, it is likely that the activation of these regions indirectly excites neurons in the amygdala.

Recently, accumulating evidence revealed that, in addition to the lateral nucleus of the amygdala (LA) [2], the central amygdala (CeA) is also crucial for fear learning [8-14]. The CeA, in particular the capsular part (CeC) [15], is strategically well situated to receive such aversive signals for the following reasons. First, the majority of CeC neurons receive nociception-related information via a direct monosynaptic pathway from the external part of the pontine lateral parabrachial nucleus (elPB) [16], which is the predominant target of the ascending nociception-specific neurons in the dorsal horn as a part of the spino-parabrachio-amygdaloid pathway [17,18]. Previous studies have demonstrated that nociceptive stimuli increase neuronal activity [19] and c-Fos immunoreactivity [20] in the elPB. Second, a number of CeC neurons are excited by noxious stimulation in the anesthetized animals, indicating these pathways are functional *in vivo* [18,21-23]. Finally, most CeC neurons receive the inputs from the indirect pathway, which is carrying highly processed polymodal signals from thalamo-cortical circuits via the basolateral amygdala (BLA) [24-30]. Importantly, these inputs from distinct nociception-related pathways converge onto single CeC neurons and show correlated synaptic potentiation following fear learning [31].

Despite these lines of evidence, it remains undetermined whether the elPB is actively involved in the regulation of nociception-induced fear learning. Here, we examined this hypothesis by 1) pharmacologically inactivating the elPB and 2) optogenetically activating the elPB-CeC circuit during training. The results indicate that the information relayed by elPB neurons is a crucial regulator for the acquisition of fear memory.

## Results

### Functional inactivation of elPB during training impairs fear acquisition

Previous studies reported that functional inactivation of the CeA during training impairs acquisition of fear conditioning [8,9]. To directly test the hypothesis that US information transmitted *via* the elPB pathway to the CeA is involved in the acquisition of fear learning, we first examined the effect of transient inactivation of the elPB during training for fear memory formation.

**Figure 1:**
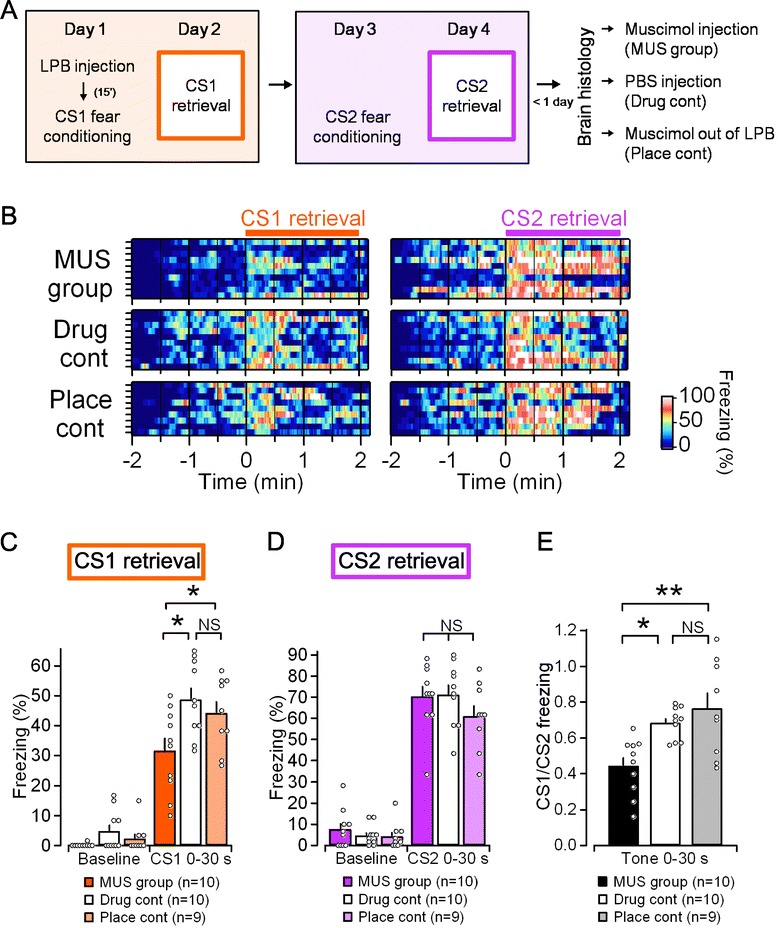
**Effects of elPB inactivation on the acquisition of auditory-conditioned fear. A**, Experimental schedule. **B**, Pseudo-color plots showing the instantaneous freezing ratio of an individual mouse during the retrieval tests following conditioning with CS1 (Day 2; left panel) and CS2 (Day 4; right panel) in the MUS group (n = 10, top), the drug control (n = 10, middle), and the placement control (n = 9, bottom). The orange and purple horizontal bars indicate the period in which the auditory cue was delivered for CS1 and CS2, respectively. **C**, **D**, Summary of freezing ratios during the first 30 s after the animal was placed in the retrieval chamber (baseline) and during the first 30 s after the onset of CS presentation. The mean ± SEM values for the three groups are shown with open circles representing the values obtained from each mouse. In the CS1 retrieval test, the freezing ratio was significantly lower in the MUS group than that in the two control groups. There were no significant differences in freezing ratio between the placement and drug-control groups **(C)**. The CS2 retrieval test revealed a significant increase in freezing ratio without any significant between-group differences **(D)**. **E**, The CS1/CS2 freezing ratio was calculated as CS1 freezing time divided by CS2 freezing time during the first 30 s after the onset of CS presentation in individual mice. The CS1/CS2 freezing ratio was significantly lower in the MUS group than that in the control groups. There were no significant differences in CS1/CS2 freezing ratio between placement and drug control groups (*p < 0.05, **p < 0.01, ANOVA and *post hoc* Tukey’s HSD test).

**Figure 2:**
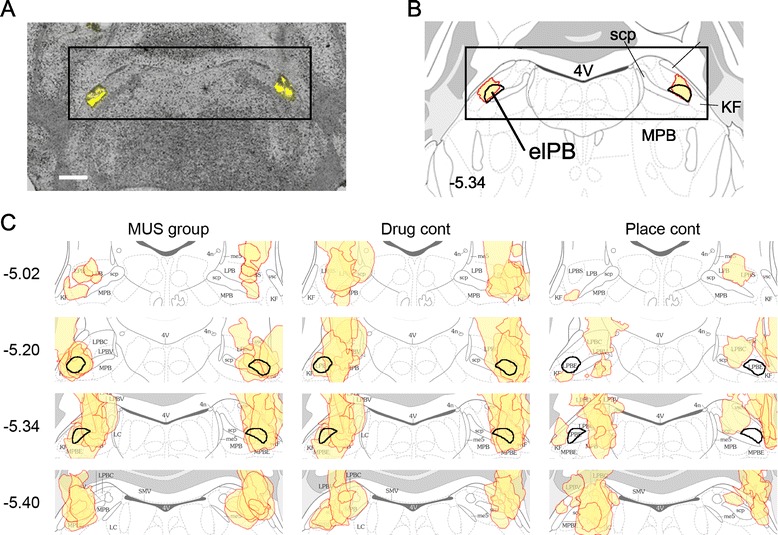
**Histological identification of injection sites. A**, Representative injection sites in the bilateral elPB identified with Lucifer Yellow are labeled as yellow (scale bar, 500 μm). **B**, Atlas from Franklin and Paxinos [15] corresponding to the level shown in **A**. The bilateral elPB are shown with black bold lines. **C**, Injection sites are indicated with red lines filled with shaded yellow for all animals used for the behavioral analyses. Each panel corresponds to the area shown with a rectangle in **A** and **B**. Numbers on the left indicate antero-posterior levels relative to the bregma. Black bold lines correspond to the bilateral elPB as shown in **B**.

Figure 1A shows the experimental design. Mice with implanted guide cannulae received infusion of the GABA_A_ agonist muscimol (MUS; 0.25 nmol, 0.1 μl per side) or an equal volume of phosphate-buffered saline (PBS), and were subjected to fear conditioning 15 min later (day 1) (Figure 1A). Mice were classified into three groups (as will be shown in Figure 2); mice with accurate injections of MUS into the bilateral elPBs were called the MUS group (n = 10), mice with accurate injections of PBS into the bilateral elPBs were drug controls (n = 10), and mice that received MUS outside of the bilateral elPB were classified as placement controls (n = 9). Fear conditioning consisted of three pairings of a pure tone (CS1) with a foot shock (0.6 mA; US), and the mice were subjected to a retrieval test 24 h later. The freezing ratio during the first post-CS period of 30 s was used as a measure of fear learning. In the CS1 retrieval test on day 2, despite there being a significantly higher freezing ratio than in the pre-CS baseline period in all three groups (baseline vs. CS1: MUS group, *p* < 0.001; drug control, *p* < 0.001; placement control, *p* < 0.001; paired *t*-test), the freezing ratio was significantly lower in the MUS group than in the other two control groups (F_(2, 26)_ = 6.792, *p* = 0.004, MUS group vs. drug control, *p* = 0.004; MUS group vs. placement control, *p* = 0.036, ANOVA and *post hoc* Tukey’s HSD test, Figure 1C). There was no significant difference in the freezing ratio between the drug and placement control groups (drug control vs. placement control: *p* = 0.702, ANOVA and *post hoc* Tukey’s HSD test). These results suggest that transient functional inactivation of the elPB disrupted fear acquisition. However, it is also plausible that guide cannula placement into the elPB may have caused some damage so that the expression of fear memory was impaired, or that infusion of MUS may have caused long-lasting rather than reversible inactivation of elPB activity. To rule out these possibilities, we conducted a second fear-conditioning test using a CS with a pip tone (CS2) that was distinct from CS1 48 h after the drug or PBS infusion (Day 3, Figure 1A). We found that the second memory retrieval test using CS2 on day 4 resulted in a significant increase in the freezing ratio compared with that observed in the pre-CS baseline period (MUS group: *p* < 0.001; placement control: *p* < 0.001; drug control: *p* < 0.001; paired *t*-test) with no significant between-group differences (F_(2, 26)_ = 1.246, *p* = 0.304), indicating integrity of fear learning potency in all groups (Figure 1D). As shown in Figure 1B, which presents the color-coded freezing ratios of individual mice in the three groups during the retrieval session expressed as a moving average of 15 consecutive video frames (7.5 s), before and after the presentation of CS1 (left) and CS2 (right), the MUS group exhibited less freezing upon CS1 presentation. By contrast, there were no significant differences with CS2.

We then examined whether fear learning was disrupted only when elPB activity was inhibited at the time of training in individual animals. Figure 1E shows the freezing time in response to CS1 normalized by the freezing time in response to CS2. The CS1/CS2 freezing ratio was indeed significantly smaller in the MUS group than in the other two groups (F_(2, 26)_ = 8.182, *p* = 0.002, ANOVA. MUS group vs. drug control, *p* = 0.017; MUS group vs. placement control, *p* = 0.002; drug control vs. placement control, *p* = 0.60; *post hoc* Tukey’s HSD test) (Figure 1E). Taken together, these results suggest that bilateral elPB inactivation attenuates fear memory acquisition.

### Identification of elPB drug injection sites

After all of the behavioral experiments were completed, mice were sacrificed and the injection sites were analyzed microscopically. Representative injection sites in the bilateral elPB and the same level from the mouse brain atlas are shown in Figure 2A and B, respectively. All injection sites in the three experimental groups used for the fear conditioning experiments are collectively shown in Figure 2C. The MUS group comprised mice with accurate injections of MUS into the bilateral elPBs (n = 10, Figure 2C left). The drug control group was composed of those mice with accurate PBS injections into the bilateral elPBs (n = 10, Figure 2C middle). The placement control group comprised mice that received MUS injections outside of the bilateral elPB (n = 9, Figure 2C right).

**Figure 3:**
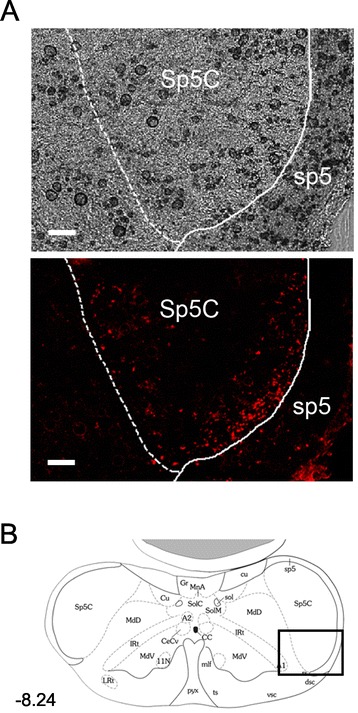
**Confirmation of the injection sites by accumulation of retrograde tracer into the spinal trigeminal nucleus caudalis (Sp5C), which sends ascending fibers to the elPB. A**, Representative photomicrographs of bright field (top) and fluorescent (bottom) images in the boxed area shown in B. Note that the injected retrograde tracer (1.25% FluoSpheres) accumulates in the superficial layer of the Sp5C 4 days after the injection, indicating that the site of injection receives projections from this region (scale bar = 100 μm). Sp5C, spinal trigeminal nucleus, caudal part; sp5, spinal trigeminal tract. **B**, Atlas corresponding to the same antero-posterior level as in A from Franklin and Paxinos [15].

To confirm that the injection site in the elPB receives projections from area known to receive primary nociceptive inputs, we next analyzed the results of retrograde tracer injections in mice after behavioral tests. For this purpose we visualized the fluorescence from the FluoSpheres (1.25%), which we had injected along with either MUS or PBS in the elPB, in the spinal trigeminal nucleus caudalis (Sp5C). The Sp5C is often considered a medullary dorsal horn because of their identical laminated organization and because it merges posteriorly without a sharp border to become the cervical spinal cord dorsal horn [32]. Additionally, nociceptive inputs preferentially target parabrachial-projecting neurons in the Sp5C rather than thalamic-projecting neurons [33]. As shown in Figure 3A and B, the injected FluoSpheres was found to be accumulated in the superficial layer of the Sp5C, confirming that the injection sites received projections from this region.

### Functional inactivation of bilateral elPB has no significant effect on the thresholds for responses to aversive and nonaversive stimuli

Impaired fear learning induced by functional inactivation of the bilateral elPB (Figure 1B,C, and E) suggests that the elPB is critically involved in the acquisition of fear memory. Another possibility is that the functional inactivation of the elPB may have simply affected the nociceptive threshold for a response to the US foot-shock. Previous reports have shown that nociceptive stimuli increase c-Fos immunoreactivity in the parabrachial nucleus [19]. Therefore, we employed the following approaches to distinguish these two possibilities. First, we evaluated immobility during fear conditioning (day 1) and examined whether the freezing ratio in the retrieval test (day 2) was correlated with immobility during conditioning. Second, we examined the effect of functional inactivation of the elPB on the thresholds for responses to aversive and nonaversive sensory stimuli, including electrical foot-shock, thermal stimulation, and mechanical stimulation, in sets of animals separate from those used for fear conditioning.

**Figure 4:**
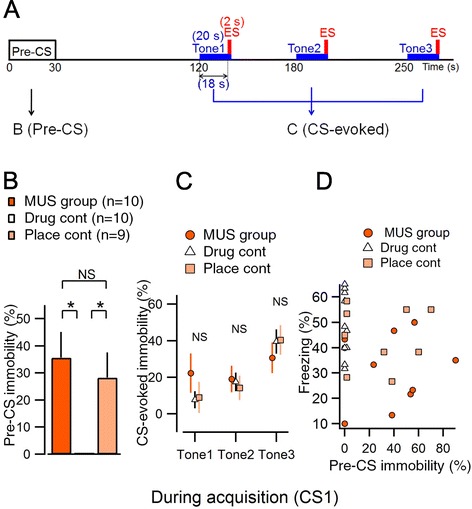
**Effects of bilateral elPB inactivation on immobility time during the training sessions. A**, Timeline of the experimental protocol. ES, electrical shock. **B**, Summary of the immobility ratio during the pre-CS period, which is the first 30 s after the animal is placed in the conditioning chamber as indicated in **A**. Both MUS and placement control groups showed significantly higher immobility than the drug control group, but there were no significant differences between them (*p < 0.05, ANOVA and *post hoc* Tukey’s HSD test). **C**, Summary of cue-evoked immobility, which is the immobility ratio during the first 18 s period of the 20 s presentation of CS (from the onset of CS until just before the onset of the 2 s US presentation period, **A)** minus the total pre-CS immobility ratio. There were no inter-group differences in cue-evoked immobility. **D**, Correlation between pre-CS immobility on day 1 and the freezing ratio on day 2. No correlation was observed (Pearson’s correlation, r = 0.217, *p* = 0.258; MUS group, r = 0.75, *p* = 0.836; drug group, r = 0.231, *p* = 0.522; placement control, r = 0.031, *p* = 0.937).

The immobility ratio was analyzed in mice during the fear conditioning session on day 1 (Figure 4A). The immobility ratio was defined in the same manner as that for the freezing ratio calculated during the training session, and the pre-CS period immobility was defined as the immobility ratio during the first 30 s after the animals were placed in the conditioning chambers after MUS or PBS injections. Both MUS and placement control groups showed significantly higher immobility than the drug control group during the pre-CS period, with no significant differences between them (ANOVA, F_(2, 26)_ = 6.263, *p* = 0.006. MUS group vs. drug control, *p* = 0.007; drug control vs. placement control, *p* = 0.039; MUS group vs. placement control, *p* = 0.777; *post hoc* Tukey’s HSD test, Figure 4B). The higher pre-CS immobility ratios observed in both the MUS and placement control groups might be attributable to an acute effect of MUS on general locomotor activity or on exploration motivation, although the precise cellular mechanisms were not determined. We also analyzed the CS-evoked immobility ratio, which was defined as the total immobility ratio during the 18 s presentation of the CS (from the onset of CS until just before the 2 s US presentation period; Figure 4A) minus the pre-CS immobility ratio. There were no inter-group differences in the CS-evoked immobility ratio (ANOVA: F_(2, 26)_ = 0.959, *p* = 0.396 for tone 1; F_(2, 26)_ = 0.141, *p* = 0.869 for tone 2; F_(2, 26)_ = 0.492, *p* = 0.617 for tone 3; Figure 4C). We then examined the correlation between the pre-CS immobility and the freezing ratio on day 2 (Figure 4D), and found no correlation between them (Pearson’s correlation, r = 0.217, *p* = 0.258); moreover, none of the three groups considered separately showed significant correlations (MUS group, r = 0.75, *p* = 0.836; drug group, r = 0.231, *p* = 0.522; placement control, r = 0.031, *p* = 0.937). These data suggest that although the MUS group showed significantly higher pre-CS immobility during the conditioning session, this immobility was not necessarily causal of the decreased freezing behavior in the retrieval test on day 2 because 1) the placement control group also exhibited higher pre-CS immobility compared with the MUS group (Figure 4B), yet the placement control group showed a freezing ratio on day 2 that was as high as that in the drug control group (Figure 1C); and 2) there was no correlation between pre-CS immobility and the freezing ratio on day 2 (Figure 4D). These results suggest that the pre-CS immobility is not predictive of fear learning in the retrieval test. Therefore, the attenuated fear learning in the MUS group is not necessarily attributable to pre-CS immobility or the immobility induced by the cue.

**Figure 5:**
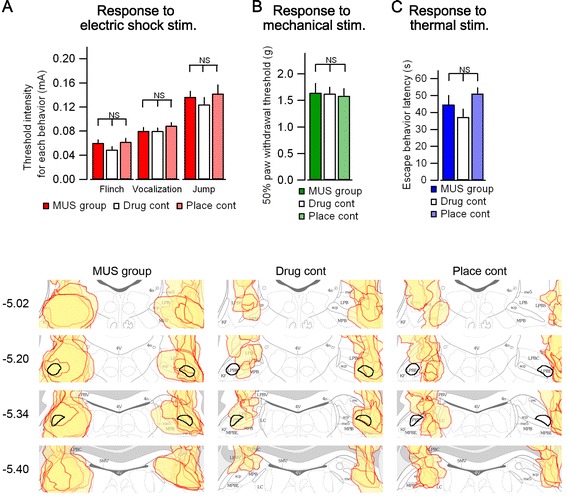
**Effects of bilateral elPB inactivation on responses to electrical foot-shock, thermal stimuli, and mechanical stimuli. A**, Summary of the stimulation thresholds for responses to flinch (left), vocalization (middle), and jump (right) in the foot-shock sensitivity test with increasing stepwise US foot-shock intensities (mA). There were no significant group differences between the MUS group (n = 12), the drug control (n = 10), and the placement control (n = 9) groups. **B**, Summary of the paw withdrawal threshold to mechanical stimuli determined by the von Frey filament test. There were no significant differences in mechanical thresholds between the groups. The threshold is shown as the average of right and left paw thresholds. **C**, Summary of the latency to thermal stimuli examined by the hot plate test. No significant group differences were observed. **D**, Histological identification of injection sites. Injection sites were indicated in the same manner as those in Figure 2C for all animals used for the stimulus sensitivity analyses. Numbers on the left indicate antero-posterior levels relative to the bregma.

We next examined the effect of functional inactivation of the elPB on the sensory threshold in response to electrical foot-shock, as well as to mechanical and thermal stimulation in sets of animals separate from those used for fear conditioning. For these experiments, we prepared three experimental groups using the same criteria as those used in the fear memory experiment (MUS group: n = 12, drug control: n = 10, placement control: n = 9, Figure 5D). We first measured the foot-shock stimulus threshold for responses of flinch, vocalization, and jump behaviors with increasing stepwise US intensities (Figure 5A). There were no significant differences between groups in terms of the thresholds for responses of flinch (F_(2, 27)_ = 1.173, *p* = 0.325), vocalization (F_(2, 28)_ = 0.584, *p* = 0.564) and jump (F_(2, 28)_ = 0.528, *p* = 0.595) behaviors. Next, we measured the paw withdrawal threshold in response to mechanical stimulation using the von Frey filament test (Figure 5B). No significant difference was observed among any of the groups (F_(2, 27)_ = 0.036, *p* = 0.965). Finally, we examined the latency to thermal stimulation using the hot plate test (Figure 5C). There was no significant difference in latency among the groups (F_(2, 27)_ = 1.791, *p* = 0.186). Six mice in the MUS group, two mice in the drug control group, and four mice in the placement control group showed no jump response within the cutoff time (60 s), suggesting that this assay system may underestimate potential differences in thermal threshold. Taken together, these results suggest that transient inactivation of the elPB has no apparent effect on mechanical and electric shock sensitivity. Therefore, our data strongly suggest that the attenuated fear learning in the MUS group is attributable to weaker fear memory acquisition rather than to reduced US sensitivity during the association.

### Activation of the elPB-CeC pathway paired with CS is sufficient to induce fear learning even in the absence of the foot-shock US

**Figure 6:**
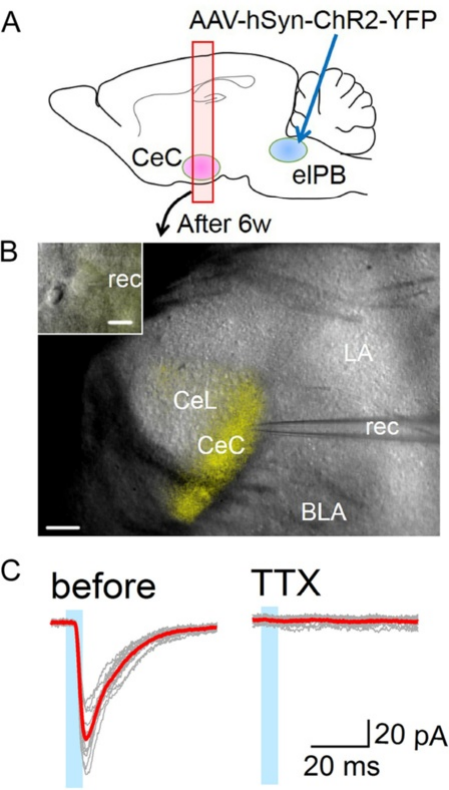
**Whole-cell patch-clamp recordings of optically evoked EPSC. A**, A schematic of the experimental approach. **B**, An oblique illumination optical image merged with YFP fluorescence (yellow) showing the recording pipette (rec). Scale bar, 100 μm. The inset shows the high magnification image around the recorded neuron (scale bar, 10 μm). CeL, the lateral division of CeA; BLA, basolateral amygdala; LA, lateral amygdala. **C**. Traces show consecutive fifteen responses (gray) evoked by optical stimulation (duration, 5 ms; every 20-s; blue box) before (left) and during (right) tetrodotoxin (TTX, 1 μM) administration. Red traces indicate the averaged waveforms.

While the above results suggest that the elPB-CeC circuit contributes to fear memory regulation, it is still unknown whether this circuit is sufficient to induce fear memory formation when paired with the CS. To directly address this unknown problem, we next employed optogenetics approach to selectively activate elPB-CeC pathway to determine whether this activation can substitute for US foot-shock. Mice received stereotaxic injections into the bilateral elPB of adeno-associated virus (AAV) expressing channelrhodopsin-2 (ChR2-YFP) to allow photoactivation of axonal terminals (Figure 6A). After 6 weeks, we observed bright ChR2-YFP expression in the axonal terminals throughout the CeC in acute amygdala slices (Figure 6B), confirming a strong elPB-CeC projection. We also confirmed that brief LED light pulses (465 nm, 5-ms duration, every 20-s) reliably evoked excitatory postsynaptic currents (EPSCs) in acute brain slices using whole-cell patch-clamp recordings, as previously reported ([34], Figure 6C).

**Figure 7:**
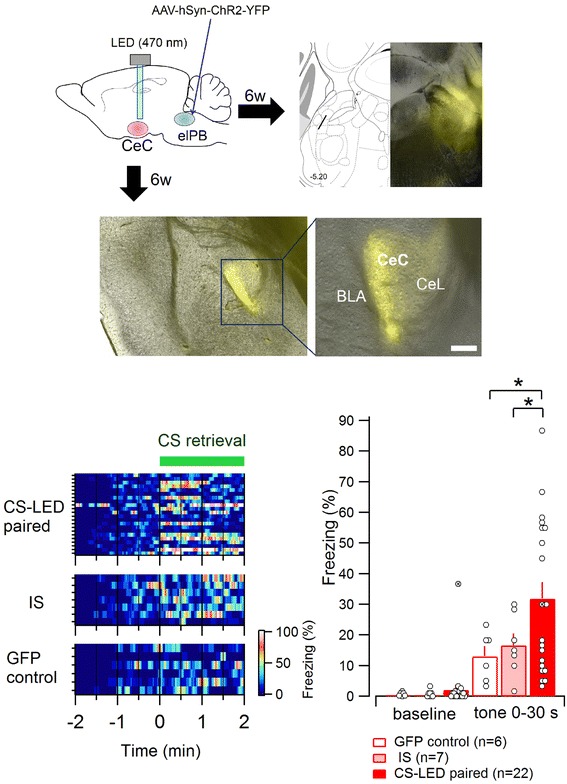
**Effects of specific activation of the elPB-CeC pathway on the auditory-conditioned fear. A**, A schematic of the experimental approach. **B**, Representative images showing bright YFP expression at the injection site (right panel) and the corresponding level of atlas [15] (left panel) in the slices prepared after completion of the behavioral experiments (6 weeks after AAV microinjection). **C**, Representative images showing bright YFP expression in the axonal terminals in the CeC region. Scale bar in the right panel, 100 μm. **D**, Pseudo-color plots showing the instantaneous freezing ratio of an individual mouse during the retrieval tests in the CS-LED paired group (n = 22, top), the immediate shock (IS) group (n = 7, middle), and the GFP control group (n = 6, bottom). The green horizontal bar indicates the period in which the auditory cue was delivered. **E**, Summary of freezing ratios during the first 30 s after the animal was placed in the retrieval chamber (baseline) and during the first 30 s after the onset of CS presentation. The mean ± SEM values for the three groups are shown with open circles representing the values for each mouse. In the retrieval test, the freezing ratio in the CS-LED paired group is significantly higher than that in both the IS group and GFP control group (CS-LED paired group vs. control, *p* < 0.05; CS-LED paired group vs. IS group, *p* < 0.05; Welch’s *t*-test followed by correction with Bonferroni-Holm method). The crossed circle in the CS-LED paired group represents an individual showing a higher freezing ratio than the others during the baseline period, yet not during CS presentation, suggesting that higher freezing ratio is not necessarily attributable to this individual. No significant differences in freezing ratio between the IS group and control group. * *P* < 0.05.

To examine the effect of terminal activation of elPB-CeC projection *in vivo*, after a minimum of 5 weeks following the AAV infection in the elPB, mice were anesthetized and bilaterally implanted just dorsal to the CeC using stereotaxic coordinates with LED cannulae that were later attached to an LED Teleoptic receiver during conditioning (Figure 7A). We confirmed bright ChR2-YFP expression in the elPB and the axonal terminals throughout the CeC at the conclusion of all the behavioral experiments (Figures 7B and C). Following to 7–8 days of postsurgical recovery, mice experienced “LED-induced fear conditioning” in which they received nine pairings of a pure tone CS (20 s) with LED stimulation (40 Hz, 5-ms pulse duration stimulation for 2 s co-terminated with the CS) (CS-LED paired group, n = 22). One group of control mice was infected with AAV carrying only GFP without ChR2 and experienced the same LED-induced fear conditioning protocol (GFP control group, n = 6). Another group of control mice expressed ChR2-YFP and experienced LED stimulation nine times immediately after being placed in the conditioning chamber and were then later exposed to the CS nine times (immediate shock; IS group, n = 7). In the IS group, the interval from the end of the last LED illumination to the onset of the first CS was 120-s in the IS group. All mice were subjected to a retrieval test 48 h later. The freezing ratio during the first post-CS period of 30 s was significantly higher in CS-LED paired group than that in both the GFP control group and the IS group (CS-LED paired group vs. GFP control, *p* <0.05; CS-LED paired group vs. IS group, *p* < 0.05; Welch’s *t*-test followed by corrections using the Bonferroni-Holm method, Figure 7D and E). These results indicate that specific activation of the elPB-CeC pathway paired with the CS is sufficient to induce associative fear memory even in the absence of a peripheral foot-shock US.

## Discussion

The elPB is a relay center involved in transmission of various types of protopathic sensory information, including temperature [35], appetite-related sense [36], and nociception [19,20]. Of these, the role of the elPB in nociceptive signaling has been most extensively described, especially in rodent models. A majority of the ascending projection neurons in the superficial layer of the spinal dorsal horn directly target the elPB [18]. This nociceptive information is then forwarded to the CeC by long-range projection neurons that directly target the CeC [16,37]. These di-synaptic connections form a “spino-parabrachio-amygdaloid pathway” involved in the processing of noxious information and in pain-related emotional complications associated with chronic pain as this pathway directly links the spinal nociceptive mechanisms and the amygdala [38,39]. Although these lines of evidence suggest that the elPB may play an essential role in the noxious US-dependent fear learning, this possibility has never been directly addressed. In the present study, we found that functional inactivation of the elPB significantly impaired fear learning (Figures 1, 2 and 3) without significantly affecting aversive and nonaversive sensory stimuli thresholds (Figures 4 and 5). These results clearly indicate that the elPB is necessary for the full expression of the foot shock-induced acquisition of fear memory. We also found that specific activation of the elPB-CeC pathway is sufficient to induce associative fear memory when the activation is paired with the CS (Figures 6 and 7). Collectively, the present results clearly demonstrated that elPB is actively involved in the fear learning.

The LA has been acknowledged as a pivotal site for associative fear/threat learning, and the generally accepted schema explains that the inputs of thalamocortical CS sensory information and noxious US information converge onto the LA to form associative plasticity [2]. The results of the present study are not necessarily incompatible with this schema. According to the recent view of ascending nociceptive signaling [40-42], the excitation of the spinal dorsal horn neurons by foot-shock would activate both spino-parabrachial and spino-thalamic pathways. Whereas the latter pathway underlies the transmission of US information to the LA, the former pathway would send this information directly to the CeC and each of these would elicit associative learning at distinct circuit levels. The freezing component that remained even after the elPB inactivation (Figures 1C and E) might be attributed to the associative learning in the LA through the thalamo-cortical US, which is essentially separate from the spino-parabrachio-amygdaloid pathway and thus was not affected by the MUS injection (It is however also possible that this incomplete suppression of freezing might be partly due to an incomplete suppression of the elPB neurons in our experiments). This interpretation is in good agreement with the recently presented series of studies providing evidence indicating that the CeA is also involved in the acquisition of fear memory [8,10-14]. It is likely that the association between the nociceptive signal via the elPB and the sensory information relayed by the BLA occurs in the CeC. Such dual-site organization for CS-US association is also supported by our observation that the both the elPB-CeC and BLA-CeC synaptic transmissions are potentiated after fear learning [31]. This dual-site system might be beneficial in assuring the optimized US-triggered learning of the CS against a variety of noxious inputs of distinct modalities.

A crucial issue for understanding the mechanisms underlying the fear learning is how noxious US triggers associative plasticity. For foot shock-induced fear learning, the US signal should originate in the peripheral nociceptors and be mediated through connections between cerebral regions composing the “pain matrix” [42] and the amygdala. Using electrolytic lesions and pharmacological inactivation, the “pain matrix” regions, including the insular cortex, the thalamic posterior intralaminar nuclei, the ACC and the PAG, have been shown to regulate fear learning and place aversion [6,7,28,43,44]. These structures, with an exception of the PAG, are activated through the spino-thalamic pathways, which mostly originate in the deep layer of the dorsal horn to which a majority of Aβ-fibers, but not C-fibers, project [18]. By contrast, most of the projection neurons in the superficial layer target the elPB. This led the authors of these reports to speculate that the remaining component of fear-related behaviors after pharmacological inactivation may be attributed to the parabrachial-mediated pathway, a possibility never being directly examined until the present study. Our results thus provided a direct and positive answer to this interpretation. On the contrary, the remaining component of freezing after intra-elPB injection of MUS observed in this study would be attributed to the spino-thalamic pathway.

The situation is a little more complicated with the PAG, which receives one third to a half of the axon collaterals of spino-parabrachial projection fibers [45], and thus shares the similar nociceptive information with the elPB. Indeed, a role of the PAG in aversive US has been demonstrated in fear learning [7,44,46]. However, the PAG has only a limited number of fibers projecting to the amygdala and thus would influence the nociceptive inputs to the amygdala through the indirect pathways involving the intralaminar thalamic nuclei, ACC, hypothalamus, locus coeruleus and the ventral tegmental area (see the discussion in Johansen et al. [7]). In this regard, despite similar contribution as a site relaying the US, the elPB and the PAG might play distinct roles in terms of the type and modality of nociceptive inputs. Whereas the PAG would be involved in more integrated aspects of the aversive signal, the elPB would be more directly linked with the spinal nociceptive mechanisms. Such dual organization of US pathways further supports the notion that the CS-US association occurs at multi-steps through the LA to the CeA and would be beneficial in ensuring the responses to a variety of aversive information.

In this study, elPB inactivation did not significantly affect the nociception-induced behaviors. The absence of changes in paw withdrawal threshold and hot plate response time may be due to the small contribution of the spino-parabrachio-amygdaloid pathway in these responses. These responses could occur mostly at the spinal network level, and would not necessarily activate this ascending pathway especially when the stimulation was brief. In other words, these reflexogenic behaviors may represent intra-spinal activity before the signal is sent to the higher brain relay centers. This interpretation is supported by the evidence that the neuropathic pain-induced elPB-CeC synaptic potentiation is abolished in animals with peripheral C-fibers lesion, despite clear manifestation of a reduced paw withdrawal threshold [47]. This suggests that the paw withdrawal reflex is independent of the elPB-CeC transmission. However, it is also possible that these measurements were not sensitive enough or saturated (for the hot plate test that was limited to 60-s). It is generally acknowledged that the ultrasonic vocalization near the 25 kHz range involves activities in the CeA and the substantia innominata [48]. The absence of an effect of elPB inactivation on the audible vocalization in the present study might be interpreted as the electrical shock not being strong enough to activate these circuits via elPB-CeC connections to trigger ultrasonic vocalization but rather the shock predominantly activated the thalamus-mediated auditory vocalization network. A recording of ultrasonic vocalization would directly address this issue.

There are two distinct pathways, the direct and the indirect pathways, to communicate peripheral nociceptive information to the amygdala. Thus, one intriguing possibility is that whereas the sensory aspect of pain is preferentially mediated via the thalamo-amygdaloid pathway, the emotional aspect of pain is more preferentially mediated via the parabrachial-amygdaloid pathway. Therefore, another possibility to explain the lack of apparent changes in the sensory threshold following elPB inactivation is that the sensory component of pain is predominantly governed by the thalamocortical pathway. Consistent with this notion, we found that optogenetic activation of the parabracial-amygdaloid pathway is sufficient to induce associative learning when paired with CS even without noxious US. This is to our knowledge the first demonstration of a non-thalamic pathway-dependent form of associative fear learning. These data suggest that the activation of the direct pathway is capable of substituting for US foot-shock. It would be interesting to examine how this direct pathway-driven associative memory is different from conventional fear memory to better understand the regulatory mechanisms of the emotional and sensory modulation of fear learning.

The elPB receives ascending fibers from the dorsal horn nociceptive neurons, and to send massive projections directly to the CeA [18]. Such an organization might be strategically beneficial for modulating the “nociception-emotion link”; a concept asserting that the perception of pain induced by a given nociceptive input might be modulated according to an animals’ emotional state, both acutely and chronically. In favor of this idea, elPB–CeC synapses are known to be highly plastic, such as in acute and chronic pain models and following fear learning [30,31,49-53] as well as in *in vitro* [53]. These synapses are also highly susceptible to neuromodulation [54]. It would be of great interest to examine the physiological roles of this synaptic plasticity to explore its clinical relevance in a future study.

## Conclusions

The results of the present study suggests that the elPB is actively involved in the regulation of fear learning. The attenuated fear learning resulting from transient inactivation of the elPB, and the enhanced fear learning resulting from selective activation of the elPB-CeC pathway, may be attributable to the disruption and the exaggeration of the nociception-emotion link, respectively. Such a regulation may play pivotal roles in survival by allowing animals to adaptively learn and avoid potentially harmful events in their environment.

## Methods

### Animals

The use of the animals was approved by the Institutional Committee for the Care and Use of Experimental Animals at the Jikei University School of Medicine (Approval No. 21-061C8). All experiments conformed to the *Guidelines for Proper Conduct of Animal Experiments* by the Science Council of Japan (2006) and to the guidelines recommended by the International Association for the Study of Pain [55]. All efforts were made to reduce the number of animals used and suffering of the animals. Male C57BL/6 J mice (CLEA Japan Inc., Tokyo, Japan) were group-housed (4 mice per cage) under a 12 h light/dark cycle, and provided with food and water *ad libitum*.

### Surgical procedures

For the pharmacological experiments, surgical implantation of guide cannulae was performed with 6-week-old mice which were anesthetized with sodium pentobarbital (45–50 mg/kg, i.p.) and placed in a stereotaxic instrument. In some cases, 0.5% isoflurane was additionally administered through a nose mask to obtain an appropriate depth of anesthesia during surgery as confirmed by mild pinching of the tail. Mice were injected with 1% xylocaine subcutaneously before head skin incision. A set of 20-gauge guide cannulae (C313GS-5/SPC, PlasticsOne, VA, USA) was implanted in the bilateral elPB according to coordinates obtained in the atlas by Franklin and Paxinos [15] (6.0 mm posterior and 1.5 mm lateral from the bregma, and 4.8 mm ventral to the skull surface) with a 20° anterior to posterior angle to avoid damaging superficial arteries during surgery. Mice were given 7 days of postoperative recovery and handled daily before commencement of fear conditioning. Microinfusions of MUS (0.25 nmol in 0.1 μl/side) or PBS (0.1 μl/side) were performed with internal cannulae (C313IS-5/SPC, 28 gauge, PlasticsOne, VA, USA) connected to 10-μl Hamilton syringes via polyethylene tubing with an infusion pump (KDS 200, KD Scientific, MA, USA) at a rate of 0.05 μl/min. The internal cannula was left in place for 2 min after the infusion. The internal cannulae for drug delivery protruded 2.3 mm beyond the tips of the guide cannulae. All the infusion sites were later verified using Lucifer Yellow (1.25%, CH dilithium salt, Sigma-Aldrich, MO, USA) and FluoSpheres (1.25%, F-8794 Molecular Probes, Thermo Fisher Scientific, MA, USA) which were co-injected with MUS or PBS.

For the optogenetics experiments, similar surgical procedures were conducted as described above, except that 4-week-old mice were used for viral injection with an adeno-associated virus (AAV5) encoding channelrhodopsin (ChR2) fused to YFP under the control of synapsin promoter (AAV5-hSyn-ChR2(H134R)-eYFP; University of Pennsylvania Vector Core) or a control virus carrying only GFP (AAV5-hSyn-eGFP). Targeted microinjection of the virus (0.25-0.5 μl) were made into the bilateral elPB (6.2 mm posterior to bregma, 1.5 mm lateral to midline, and 4.4 mm ventral to the cortical surface, with a 20° anterior to posterior angle to avoid damaging superficial arteries during surgery) using a Hamilton microsyringes (1701RN Neuros Syringe, 33 G, 10 μl) with an injection speed (50 nl/min) controlled with a microsyringe pump (UltraMicroPumpII with SYS-Micro4 Controller, UMP2, UMC4, World Precision Instruments, Florida, USA). The injection syringes were left in place for 10 minutes before withdrawal. After 5 weeks, a second surgical procedure was conducted for bilateral LED cannula placement. The bilateral LED cannula unit was composed of an LED (blue, 470 nm) body part attached to a dual cannulae unit, which is composed of dual optic fibers with a 0.25 mm diameter, 4.0 mm length, and 5.0 mm space interval (TeleLCD-B-4-250-5, Bio Research Center, Tokyo, Japan). The LED cannulae unit was inserted targeting the CeA according to coordinates obtained in the atlas by Flanklin and Paxinos [15] (1.4 mm posterior to bregma, 2.5 mm lateral to midline) stereotaxically, and secured to the skull with dental cement (GC Fuji I, GC Corporation, Tokyo, Japan).

### Fear conditioning

Fear conditioning experiments were conducted as previously described [31]. For the pharmacological inactivation experiments, mice received injections of either PBS or MUS into the elPB through the guide cannulae in the home cage on the day 1. After 15 min, mice were placed in a conditioning chamber (170 mm width × 100 mm depth × 100 mm height, 200 Lux, 50 dB background white noise) surrounded by a sound-attenuating chamber (CL-M3, O’Hara & Co., Ltd., Tokyo, Japan). Following to 2-min observation period, the mice were conditioned with three pairings of a 20 s CS tone (CS1: pure tone, 10 kHz, 65 dB) that terminated concurrently with a foot-shock (US; 0.6 mA, 2 s) (see Figure 1A and 4A). Foot-shocks were delivered to the floor grid of the chamber through a shock generator (O’Hara & Co., Ltd, Tokyo, Japan). The first CS was delivered 120 s after the animal was placed in the chamber, and the inter-trial intervals were 40 s and 50 s. A retrieval trial was performed 24 h later (day 2) with delivery of CS1 in a retrieval chamber (with white acrylic plate walls scented with peppermint odor; 50 Lux, 60 dB background white noise). The CS1 presentation began 120 s after the mice were placed in the chamber, and lasted for 120 s. Mouse behavior was captured using a digital camera at 2 frames/s and freezing behavior was analyzed using Time FZ1 software (O’Hara & Co., Ltd), a package based on NIH Image. The movement of the mouse was detected by pixel-to-pixel subtraction between two subsequent frames and the behavior at each frame was defined as “freezing” (defined for retrieval tests; day 2 and 4) or “immobility” (defined for conditioning; day 1 and 3) when the total number of pixels with detectable frame-to-frame difference was less than 30. The identification of freezing and immobility behavior was pre-optimized by two independent human observers using C57BL/6 J mice. On day 3, mice underwent another conditioning with a distinct CS tone (CS2: a 4 Hz pip tone consisting of a 50 ms tone and a 200 ms interval at 12.5 kHz, 65 dB) in the same manner as that for CS1, without any drug infusion (see Figure 1A). A retrieval test was performed 24 h later (day 4) with delivery of CS2 to an animal in a distinct retrieval chamber having black and white acrylic board walls washed with lemon-scented soap (55 Lux, 65 dB background white noise). Freezing behavior was measured in the same manner as that for the CS1 retrieval test. All behavioral analyses were conducted by experimenters blinded to the pharmacological manipulations, and all postmortem histological analyses were conducted by experimenters blind to the behavioral analyses.

For the optogenetics experiments, mice were recovered from the surgery for 7–8 days. One day before fear conditioning, mice were habituated with dummy Teleopto receivers (2 g, TeleDummy, Bio Research Center) attached to the bilateral LED cannula unit for 1 h. On the conditioning day, an infrared light-driven wireless LED unit Teleopto receiver (2 g, TeleR-2-P, Bio Research Center) was attached to the bilateral LED cannulae unit in the home cage, and mice were placed into the conditioning chamber (200 Lux, 50 dB background white noise) as the pharmacological experiments described above. After 2-min period, mice received nine pairings of a 20 s CS tone (10 kHz, 65 dB) that terminated concurrently with a 2-s LED illumination (5 ms, 40 Hz, 4.5 mW) controlled by an infrared light-driven remote controller (Teleopto remote controller, Bio Research Center) placed on the inside wall of the sound-attenuating chamber (CL-M3, O’Hara & Co., Ltd., Japan). The delivery of light pulses was precisely controlled by a programmable stimulator (Master-8, A.M.P. Instruments LTD) and Time FZ1 software (O’Hara & Co., Ltd). No US foot-shock was delivered through the floor grids for these experiments and the shock generator power was off. The optical intensity was measured before each session to ensure consistent output of 1 mW or above measured at a tip of an optic fiber. The inter-trial intervals for the nine pairings were pseudo-randomly selected, ranging from 40 to 480 s, as described previously [31]. Two days later, a retrieval test was performed with CS in a retrieval chamber as described above, during which period the mice were wearing dummy receiver attached to their heads.

### Evaluation of nociceptive responses

Flinch, vocalization, and jump threshold to foot-shock were evaluated in the conditioning chamber used for auditory fear conditioning in the 200 Lux and 50 dB background white noise environment. Mice received bilateral elPB injections of either PBS or MUS, and 15 min later they were placed individually in the chamber. After a 90-s period of habituation, foot-shocks with increasing intensities were given in a stepwise manner (from 0.04 to 0.24 mA, in 0.04 mA steps). The time gap between shocks was 30 s, shock duration was 1 s, and each animal was tested only once. Any detectable reaction to the shock, typically a moving-back response was regarded as flinch behavior, and the flinch threshold was defined as the lowest shock intensity that elicited a flinch. The vocalization threshold was defined as the lowest shock intensity that elicited an audible vocalization, which was captured using an auditory microphone (300–9,000 Hz: ECM-AW3: Sony, Tokyo, Japan) inside the sound-attenuating chamber, digitized with a converter (24 bit, 96 kHz: Sound Blaster Premium HD, Creative, Singapore) and stored on a PC (Lenovo Japan, Tokyo). The jump threshold was defined as the lowest shock intensity that elicited jump behavior with simultaneous removal of both hindpaws from the grid. In this manner, the thresholds for the flinch, vocalization, and jump responses in milliamperes were defined for each mouse. The paw withdrawal threshold to mechanical stimuli was evaluated using a series of von Frey filaments (North Coast Medical, Inc., Gilroy, CA, USA) of different rigidity (0.02–2.0 g) as previously described [30,31]. Each mouse was placed on a metal mesh floor, and allowed to habituate in a 500 ml glass beaker placed upside down for 30 min prior to the experiments. The 50% threshold was estimated using the up-and-down method [56]. The mechanical threshold was determined as the average of both hindpaw measurements per mouse. Following the von Frey filament test, thermal nociceptive response was evaluated by recording the latency to jump behavior on a hot plate (54.5°C, T2CT-1108, Bioseb, Vitrolles, France) within 40 min after the drug injection. The trial was terminated at 60 s, regardless of the response, to prevent potential tissue damage*.* All behavioral analyses were conducted by an experimenter blind to the pharmacological manipulations.

### Behavioral data analysis

The statistical significance of between-group differences in behavioral data was analyzed using either paired t-tests, one-way ANOVA followed by Tukey’s HSD tests, or Welch’s *t*-test followed by corrections using the Bonferroni-Holm method. Differences were considered statistically significant at *p* < 0.05.

### Histology

Within 24 h after the final behavioral test, all the mice were anesthetized with isoflurane (5%) and sacrificed for histological analysis. For the pharmacological behavior experiments, the brains were removed and blocks containing the elPB and the caudal part of the Sp5C in the brainstem were prepared in ice-cold PBS. The blocks were frozen after embedding into freezing compound (O.C.T. Compound, Sakura Finetek, Tokyo, Japan), and frozen specimens were sectioned on a cryostat (Leica, CM 1850) and mounted on glass slides. The slices were dried and examined under a fluorescent microscope (Keyence, BZ-9000). Pontine tissue slices were sectioned at a thickness of 30 μm for identification of bilateral elPB injection sites. Brainstem slices were sectioned at a thickness of 60 μm for observation of FluoSpheres in the Sp5C. All histological analyses were conducted by an observer blind to the behavioral experiments.

For the optogenetics behavioral experiments, after all the behavior tests, mice were anesthetized and the brains were extracted and post-fixed in 4% paraformaldehyde overnight at 4°C. The brain blocks were embedded in 1.6% low melting point agarose surrounded by 5% agar in cold phosphate-base buffer (pH 7.4). The agar blocks were secured on the cutting stage of a vibrating tissue slicer (PRP7; Dosaka EM, Japan) and sectioned transversely into 100-μm-thick sections. The sections were mounted on glass slides to obtain images of ChR2-YFP for viral vector injection sites and the projection sites in the CeC using Olympus BX63 fluorescent microscope.

### Electrophysiology

Coronal brain slices of 400-μm thickness containing the amygdala were prepared 6 weeks after the AAV microinjection into the elPB according to the method established in our laboratory ([34], Sugimura et al., *in submission*). The block of forebrain containing the CeA was dissected at the midline in an ice-cold cutting solution composed of (in mM) 2.5 KCl, 0.5 CaCl_2_, 10 MgSO_4_, 1.25 NaH_2_PO_4_, 2 thiourea, 3 sodium pyruvate, 92 N-Methyl-D-glucamine, 20 HEPES, 12 N-acetyl-L-cysteine,25 D-glucose, 5 L-ascorbic acid and 30 NaHCO_3_ equilibrated with 95% O_2_ plus 5% CO_2_ (pH ~7.4; osmolality, approximately 280 mOsm/kg). The dissected hemisphere was secured on the cutting stage of a vibrating blade slicer (VT1200S, Leica) with the rostral end upwards. The slices were first incubated in a holding chamber with a constant flow of cutting solution at 34°C for 15 to 20 min. The slices were kept at room temperature (20–25°C) in the standard artificial cerebrospinal fluid (ACSF) composed of (in mM) 125 NaCl, 3 KCl, 2 CaCl_2_, 1.3 MgCl_2_, 1.25 NaH_2_PO_4_, 10 _D_-glucose, 0.4 _L_-ascorbic acid and 25 NaHCO_3_ (pH 7.4 bubbled with 95% O_2_ + 5% CO_2_; osmolality, approximately 310 mOsm/kg) until the electrophysiological recording. Each slice was transferred to a recording chamber (approximately 0.4 ml volume) and fixed with nylon grids to a platinum frame. The slice was submerged in and continuously superfused at a rate of 1.5 – 2 ml/min with standard ACSF. Picrotoxin (100 μM) was added to the ACSF to isolate EPSCs. Neurons in the CeC were visually identified under an upright microscope (BX-51WI, Olympus) with oblique illumination. Epifluorescence images of YFP were captured using a CCD camera (IR-1000, DAGE-MTI) and stored digitally on a computer. Whole-cell membrane current was recorded from visually identified CeC neurons surrounded by YFP-positive terminals. Patch-clamp electrodes were made from borosilicate glass pipettes (1B120F-4; World Precision Instruments). The composition of the internal solution was (in mM) 120 potassium gluconate, 6 NaCl, 1 CaCl_2_, 2 MgCl_2_, 2 ATP Mg, 0.5 GTP Na, 12 phosphocreatine Na_2_, 5 EGTA and 10 HEPES hemisodium (pH 7.2 as adjusted with KOH; osmolarity, approximately 310 mOsm/kg). The tip resistance of the electrode was 4–8 MΩ. The ChR2 channels were activated using high power LED illumination system (465 nm; 8.4-12.7 mW(mm)^−2^; LEX2-B, Brainvision, Tokyo, Japan) controlled by Master 8 (A.M.P. Instruments LTD; pulse duration, 5 ms; every 20 sec). Membrane current was recorded using Axopatch 200B amplifier (Molecular Devices, Sunnyvale, CA, USA), filtered at 2 kHz and digitized at 10 kHz with a 16-bit resolution using a PowerLab interface (AD Instruments). All recordings were made at room temperature (20–25°C). All compounds were purchased from Nacalai Tesque or Sigma.

## Abbreviations

CeA: Central nucleus of the amygdala
CeC: The capsular division of CeA
elPB: External part of the lateral parabrachial nucleus
BLA: Basolateral nucleus of the amygdala
CS: Conditioned stimulus
US: Unconditioned stimulus
MUS: Muscimol
ChR2: Channelrhodopsin-2
AAV: Adeno associated virus
IS: Immediate shock
